# R97 at “Handlebar” Binding Mode in Active Pocket Plays an Important Role in Fe(II)/α-Ketoglutaric Acid-Dependent Dioxygenase *cis*-P3H-Mediated Selective Synthesis of (2S,3R)-3-Hydroxypipecolic Acid

**DOI:** 10.3390/molecules28041854

**Published:** 2023-02-15

**Authors:** Jiaojiao Guan, Yilei Lu, Zixuan Dai, Songyin Zhao, Yan Xu, Yao Nie

**Affiliations:** 1 Lab of Brewing Microbiology and Applied Enzymology, School of Biotechnology and Key Laboratory of Industrial Biotechnology of Ministry of Education, Jiangnan University, Wuxi 214122, China; 2 State Key Laboratory of Food Science and Technology, Jiangnan University, Wuxi 214122, China; 3 Suqian Industrial Technology Research Institute of Jiangnan University, Suqian 223814, China

**Keywords:** L-pipecolic acid, dioxygenase, hydroxypipecolic acid, “handlebar” mode, site-directed saturation mutagenesis

## Abstract

Pipecolic acid (Pip) and its derivative hydroxypipecolic acids, such as (2S,3R)-3-hydroxypipecolic acid (*cis*-3-L-HyPip), are components of many natural and synthetic bioactive molecules. Fe(II)/α-ketoglutaric acid (Fe(II)/2-OG)-dependent dioxygenases can catalyze the hydroxylation of pipecolic acid. However, the available enzymes with desired activity and selectivity are limited. Herein, we compare the possible candidates in the Fe(II)/2-OG-dependent dioxygenase family, and *cis*-P3H is selected for potentially catalyzing selective hydroxylation of L-Pip. *cis*-P3H was further engineered to increase its catalytic efficiency toward L-Pip. By analyzing the structural confirmation and residue composition in substrate-binding pocket, a “handlebar” mode of molecular interactions is proposed. Using molecular docking, virtual mutation analysis, and dynamic simulations, R97, E112, L57, and G282 were identified as the key residues for subsequent site-directed saturation mutagenesis of *cis*-P3H. Consequently, the variant R97M showed an increased catalytic efficiency toward L-Pip. In this study, the *k*_cat_/*K*_m_ value of the positive mutant R97M was about 1.83-fold that of the wild type. The mutation R97M would break the salt bridge between R97 and L-Pip and weaken the positive-positive interaction between R97 and R95. Therefore, the force on the amino and carboxyl groups of L-Pip was lightly balanced, allowing the molecule to be stabilized in the active pocket. These results provide a potential way of improving *cis*-P3H catalytic activity through rational protein engineering.

## 1. Introduction

Pipecolic acid is a chiral cyclic non-proteinaceous amino acid and a proline homolog; it is found in many natural and synthetic bioactive molecules [1]. Its derivative, viz. hydroxypipecolic acid, is widely distributed in nature, and is found in some antibiotics, terpenoids, and alkaloids; it is also an important building molecule used in the synthesis of chiral drugs, as well as in other fields [1,2]. For example, (2S,5S)-5-hydroxypipecolic acid is used as the precursor for the synthesis of an effective and irreversible β-lactamase inhibitor MK7655 [3], (2S,4R)-4-hydroxypipecolic acid has been found to be present in ulleungamides [4], (2R,4R)-4-hydroxypipecolic acid has been found to be present in damipipecolin, which was found to regulate the activity of serotonin receptor in vitro [5], and (2S,3R)-3-hydroxypipecolic acid (*cis*-3-L-HyPip) has been shown to couple with amine to form a precursor of (-)-tetrazomine, which has a significant antibacterial activity and strong cytotoxicity against P388 leukemia cells [6]. In addition, *cis*-3-L-HyPip has been used as the building block in the biosynthetic pathway of GE81112, which inhibits the formation of translation initiation complex as a translation inhibitor [7].

*cis*-3-L-HyPip can be synthesized in several ways: racemization followed by enzymatic resolution; diastereoselective methods, which typically start with natural or unnatural amino acids (serine and its derivatives, glutamic acid, or proline), carbohydrates (glyceraldehyde or D-glucose), optically active α-hydroxycarboxylic acids, or 1,2-aminoethanol or sulfoxide, as precursors [8,9,10]. However, dangerous and toxic chemicals are usually used in these synthesis processes, ones which yield toxic by-products and lead to environmental problems. In addition, they also involve the disadvantages of poor selectivity and high energy consumption. On the other hand, enzymatic synthesis is a more feasible alternative because of its high catalytic efficiency, high selectivity, low energy consumption, and environmental friendliness. Due to these advantages, enzymatic synthesis has been widely employed in the preparation of high value-added compounds.

**Figure 1:**
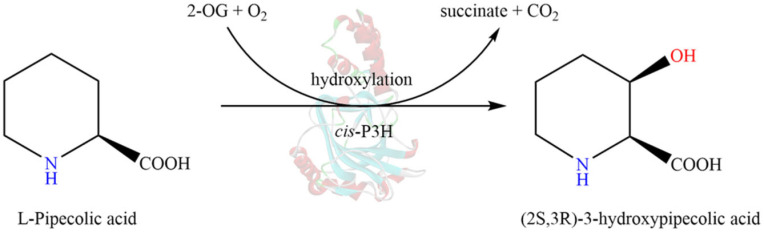
Hydroxylation of L-Pipecolic acid catalyzed by Fe(II)/2-OG-dependent dioxygenase *cis*-P3H. The involved hydroxyl group was labeled in red; the imino groups were labeled in blue.

Fe(II)/α-ketoglutaric acid (Fe(II)/2-OG)-dependent dioxygenases have become the largest family of non-heme oxidases known to catalyze a variety of oxidative transformations, such as hydroxylation, halogenation, cyclization, ring expansion, and other reactions [11]. They are also involved in the synthesis of some important secondary metabolites, such as β-lactam antibiotics, flavonoids, alkaloids, and gibberellins [12]. Among these reactions, the most extensively studied reaction for which the reactivity has been determined is substrate hydroxylation [13]. During the catalytic reaction, Fe(II) and key amino acid residues connect via coordination bonds to form a catalytic active center [14]. With the formation of hydroxylation products, α-ketoglutaric acid undergoes oxidative decarboxylation to produce succinic acid. Notably, only certain enzymes belonging to this family have been identified to catalyze the conversion of L-pipecolic acid (L-Pip) to *cis*-3-L-HyPip, including L-proline *cis*-3-hydroxylase (*cis*-P3H, type I) from *Streptomyces* sp. strain TH1 (Figure 1) [15,16], L-proline *cis*-4-hydroxylase (*cis*-P4H) from *Sinorhizobium meliloti* [15], and GetF from *Streptomyces* sp. L-49973 [17]. In addition, among the known enzymes with the activity and selectivity required to convert L-Pip to *cis*-3-L-HyPip, *cis*-P3H has limited catalytic activity toward L-Pip.

The catalytic activity of the above-mentioned dioxygenases toward the unnatural substrate L-Pip illustrates the ability of these enzymes to catalyze the conversion of L-Pip to *cis*-3-L-HyPip. Focusing on the hydroxylation required for the catalytic reaction, herein we have evaluated some enzymes belonging to the Fe(II)/2-OG-dependent dioxygenase family. Given the limited catalytic activity of *cis*-P3H toward L-Pip, we engineered the wild type to improve its ability to catalyze the formation of *cis*-3-L-HyPip based on structurally conformational analysis. Using structural modeling, molecular docking, dynamic simulation, and virtual mutation, key residues around the substrate were selected for site-directed saturation mutagenesis. The mutation R97M increased the catalytic efficiency of *cis*-P3H toward L-Pip. Our findings verified the potential of proline hydroxylases to catalyze the hydroxylation of L-Pip and the feasibility of *cis*-P3H modification.

## 2. Results and Discussion

### 2.1. Catalytic Performance of Dioxygenase Candidates

Twelve Fe(II)/2-OG-dependent dioxygenases with the potential to catalyze amino acid hydroxylation were collected (Table 1). In addition to proline hydroxylases, several enzymes from other families with hydroxylation function were also included. The enzymes were heterologously expressed in *E. coli* BL21 (DE3) with pET-28a as the vector, and the recombinants were cultured at 17 °C in LB medium. Almost all proteins could be heterologously expressed in their soluble forms (Figure S1, Supplementary Materials). The bands observed upon SDS-PAGE analysis were consistent with the predicted molecular weights (*trans*-P4H, 29.71 KDa; *Ka*PH1, 29.02 KDa; *Ka*PH2, 29.48 KDa; *cis*-P4H, 32.03 KDa; *cis*-P3H, 33.15 KDa; VioC, 39.43 KDa; AsnO, 35.98KDa; AsnOD241N, 38.90 KDa; LdoA, 30.62 KDa; IDO, 27.84 KDa; NkLH4, 42.08 KDa). The results of enzyme activity assay are shown in Figure S2.

**Figure 2:**
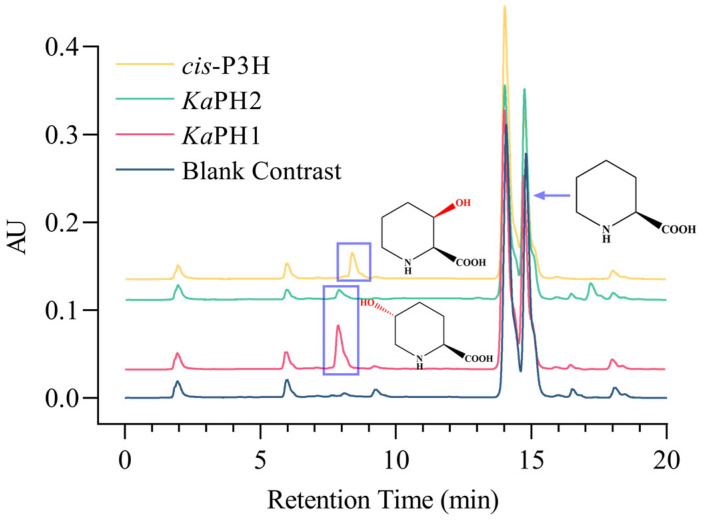
Recombinant proteins with catalytic activity toward the substrate, L-Pip. The blank contrast was the same as the experimental group except for the addition of buffer instead of enzyme. The retention time for the product of interest of reactions catalyzed by *Ka*PH1 and *Ka*PH2 was around 7.890 min, and that for the reaction catalyzed by *cis*-P3H was around 8.498 min.

Subsequently, we investigated the catalytic activity of these enzymes using L-Pip as the substrate. This was achieved using crude enzymes under the conditions of an initial enzyme activity determination system. The hydroxylases other than proline hydroxylases did not show catalytic activity toward L-Pip. In addition to *cis*-P3H, the proline hydroxylases *Ka*PH1 and *Ka*PH2 had obvious peaks near the retention time of 7.890 min, indicating their catalytic activity toward L-Pip; the catalytic activity of *Ka*PH1 was higher than that of *Ka*PH2 (Figure 2). Due to the different regioselectivity of the enzymes, *cis*-P3H catalyzed the formation of the expected product (2S,3R)-3-hydroxypipecolic acid (*cis*-3-L-HyPip), while *Ka*PH1 and *Ka*PH2 catalyzed the formation of (2S,5R) -5-hydroxypipecolic acid. Therefore, the retention times of the hydroxylated products were different. This result also indicated that proline hydroxylases have the potential of catalyzing L-Pip hydroxylation and even being used as versatile biocatalysts [15].

The results obtained from HPLC and LC-MS analyses showed the presence of mass spectrometric peaks consistent with the molecular weight of the hydroxylated products, thereby confirming the formation of (2S,5R)-5-hydroxypipecolic acid after hydroxylation of L-Pip catalyzed by *Ka*PH1; notably, *cis*-P3H yielded the desired product, viz. *cis*-3-L-HyPip (Figures S3 and S4). Our findings verified the potential of proline hydroxylases to catalyze the hydroxylation of L-Pip; more such enzymes may be discovered by mining the homologous enzymes of proline hydroxylases.

### 2.2. Structural Analysis of cis-P3H

**Figure 3:**
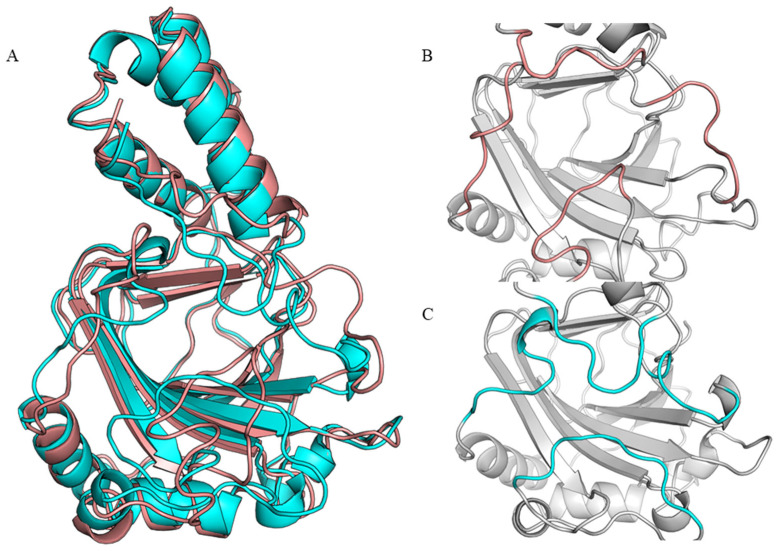
Structural analysis of *cis*-P3H: (**A**) structural alignment for *cis*-P3H: homology modeling result based on *cis*-P3H (type II) (salmon) and AlphaFoldDB (P96010, cyan); (**B**) loop region obtained from homology modeling; and (**C**) loop region of P96010.

To improve the hydroxylation activity of *cis*-P3H toward L-Pip, structural analysis of *cis*-P3H was conducted for further structure-based engineering of the enzyme. However, the X-ray crystal structure of the target enzyme *cis*-P3H (type I) is not available. In recent years, the highly revolutionary artificial intelligence network, AlphaFold, has received researchers’ attention and has been used widely. It can not only predict the structure of many proteins with high accuracy, but also provides information regarding its prediction accuracy [28]. Herein, we used the primary amino acid sequence of the target enzyme, *cis*-P3H, as input and selected the structural model in the AlphaFold protein structure database (AlphaFoldDB: P96010) (Figure 3A). Further, the structure of *cis*-P3H (type II) from *Streptomyces* sp. TH1 has been resolved (PDB ID: apo-form, 1E5R; holo-form with iron, 1E5S) and it has a sequence similarity with *cis*-P3H (type I) (75.86% identity) (Figure S6) [29]. Compared with the homology modeling result based on *cis*-P3H (type II), the loop region of P96010 in AlphaFold database had higher confidence. We also performed structure alignment and found that the loop region obtained from homology modeling was “open”, while that of P96010 was “closed,” which was more in line with the substrate-binding conformation in catalysis. The loop region was lid-like, which facilitated the substrate to anchor at the active center (Figure 3B,C). Therefore, we ultimately chose the AlphaFold structure as the model for subsequent experiments.

**Figure 4:**
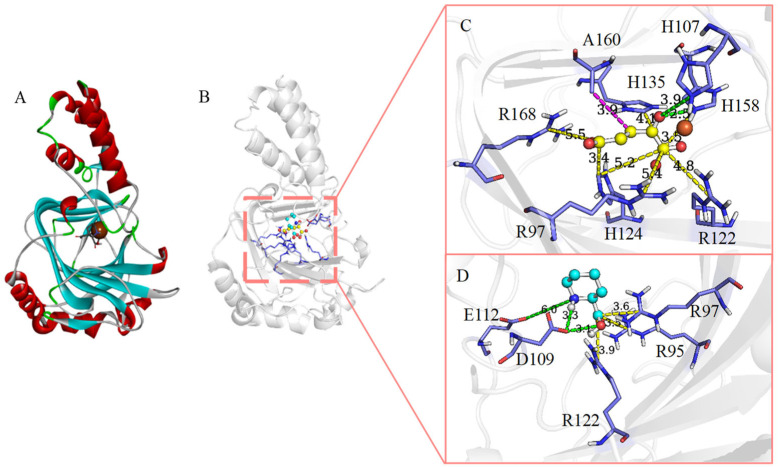
Structural analysis of substrate-binding complex. (**A**) The overall structure of *cis*-P3H (type I, AlphaFoldDB: P96010). The structure shows a double-stranded β-helix core fold (cyan), which is sandwiched between the N-terminal and C-terminal α-helical domains (red). (**B**) The molecular docking result of *cis*-P3H and L-Pip. (**C**) Amino acid residues interacting with 2-OG (yellow); hydrophobic interaction (magenta dashed line), hydrogen bond (green dashed line), and salt bridge (yellow dashed line). (**D**) A “handlebar” mode for binding of substrate L-Pip (cyan).

Before molecular docking of the substrate, we examined the amino acid residues for binding with Fe^2+^ and 2-OG to confirm the positions of Fe^2+^ and 2-OG in the active pocket. The overall structure of *cis*-P3H (type II) was very similar to that of MlP4H from *Mesorhizobium loti* [30]. Moreover, the structure of MlP4H contained Co^2+^ as a substitute for Fe^2+^, 2-OG as a cofactor, and L-Pro/L-Pip as the substrate (PDB ID: 4P7W, 4P7X). This provided us a basis for confirming the positions of Fe^2+^ and 2-OG, and to dock the target molecule L-Pip onto the active site of the model structure. Fe^2+^ was observed to be coordinated by amino acid residues H107, D109, and H158, forming the conserved iron binding motif (HxD/H motif) among Fe(II)/2-OG-dependent dioxygenases [29,31]. The carbonyl and C-1 carboxyl groups of 2-OG were bound to the metal active center Fe^2+^, and its anchoring involved the interaction between C-5 carboxyl group and side-chains of R168 and H124 (Figure 4C) [29]. In terms of substrate L-Pip anchoring, a “handlebar” binding mode was proposed. We observed the presence of three basic amino acids, viz., R95, R97, and R122, whose side chains pointed to the active center iron and formed salt bridges with the carboxyl group of L-Pip to help substrate anchoring [29]. These three amino acid residues were found to be located in the important structure of the double-stranded β-helix core fold (DSBH). It has been speculated that the β-chain in the DSBH fold can provide support and help in the selectivity of active sites and specific binding sites toward the substrate [32]. In addition, the amino group of L-Pip interacted with the acidic amino acids D109 and E112 by hydrogen bonding (Figure 4D) [30]. This helped in further exploration of substrate anchoring interactions and subsequent selection of key residues.

### 2.3. Site-Directed Saturation Mutagenesis of cis-P3H

**Figure 5:**
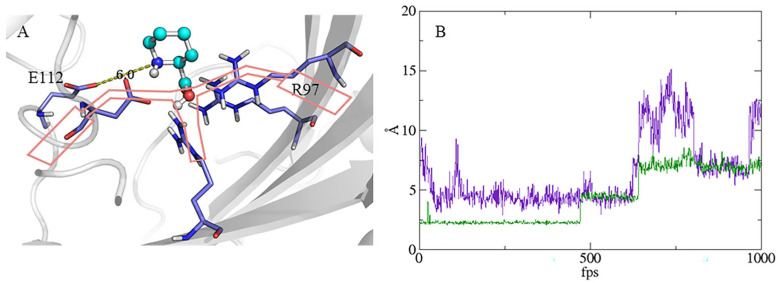
Prediction of potential sites for site-directed saturation mutagenesis. (**A**) Key residues related to substrate anchoring; E112 is 6 Å away from the amino group of L-Pip. (**B**) The dynamic changes of the distance between Y33 in the left flexible region and G282 (purple curve). The dynamic changes of the distance between Fe^2+^ and the amino acid residue H107 that constituted the iron binding motif (green curve).

The virtual saturation mutagenesis was performed on the residues within 5 Å from the L-Pip ligand and residue E112 using Discovery Studio (Figure S7). Virtual mutation analysis can reveal the energy change after mutation to determine the affinity of amino acids and substrates after the mutation [33]. The prediction of these mutation targets can guide us in making reasonable mutations. Considering the “handlebar” binding mode mentioned above, R95, R97, and R122 showed salt bridge interaction with L-Pip, and D109 showed hydrogen bond interaction with L-Pip. Compared with D109, the distance between E112 and L-Pip was relatively greater, and its interaction with L-Pip was limited [30]. In this case, the force on both sides of the “handlebar” was unbalanced, thereby making the substrate unable to be stably anchored in the active pocket (Figure 5A). Compared with the E112 on the left side of the “handlebar” with weak interaction with the substrate, the R97 on the right side of the “handlebar” formed a strong salt bridge with the substrate [30]. Thus, the interactions on the two sides of the “handlebar” might be unbalanced. In order to balance the forces on both sides of the “handlebar”, R97 in the equal position as E112 would be a preferable mutation site rather than R95 and R122 in the active pocket. Therefore, the key residue R97 was selected to weaken the interaction with the carboxyl group of L-Pip, while the key residue E112 was selected to enhance the interaction with the amino group of L-Pip [30]. In this way, the “handlebar” was stabilized, and the interaction force of the substrate was slightly balanced. Besides, the results of the virtual mutation analysis also predicted that the mutations at these two sites would enhance the affinity to the substrate. We also carried out molecular dynamics simulation of the docking results and subsequent trajectory analysis. We found that the active site was covered by loop regions, and studies have shown that this lid structure facilitates substrate anchoring at the active center [34]. Trajectory analysis results showed that Y33 in the left flexible region squeezed Fe^2+^ out of the active center. The flexible region contained six consecutive acidic residues (EEEYDE), some of which interacted with other residues or substrate and contributed to controlling the entrance of substrates [29,35]. It was speculated that the mutation of Y33 might have an influence on enzyme activity. Therefore, Y33 was not directly considered as a candidate substitution residue. Y33 squeezing Fe^2+^ out of the active center was a phenomenon observed by the trajectory analysis through visual analysis software VMD. The distance between Y33 and G282 was stable for a period of time within 5 Å, and the iron-binding motif was also in a steady conformation (Figure 5B). It was speculated that Y33 and G282 might have an interaction at this time. However, when the distance between Y33 and G282 fluctuated greatly, the distance between Fe^2+^ and H107 which constituted the iron-binding motif obviously varied. At this stage, trajectory analysis also indicated that Fe^2+^ was far from the active center and the conservative iron-binding motif collapsed. Therefore, G282 and L57, which was opposite to G282 in the lower flexible region, were also considered to be selected for mutations. It was expected that their mutations could make the lid structure more stable. Therefore, four residues, namely R97, E112, L57, and G282, were considered to be the likely candidates for subsequent site-directed saturation mutagenesis.

The saturation mutagenesis library for the above-mentioned four key amino acid residues was constructed. The mutants were expressed in *E. coli* BL21(DE3) using pET-28a vector, and the supernatant of the disrupted solution was allowed to react with the enzyme activity assay system. Only R97M and R97F mutants showed hydroxylation activity, while mutations at the other three amino acid residues individually did not show any apparent activity. Through sequence conservation analysis, E112 was highly conserved in the dioxygenase family. In addition, E112 formed molecular interactions involving salt bridge and hydrogen bond with R279 on the “lid” structure, while R279 had a close connection with D109, which composed the iron-binding motif [29]. Therefore, the mutation of E112 might indirectly affect the stability of the iron-binding motif, resulting in the loss of enzyme activity. Although L57 and G282 were not highly conserved amino acids, they were located in the loop region, which could form a lid on the active site and ‘lock’ the enzyme-substrate complex during the catalytic process [29]; mutations at these residues might affect this function. Therefore, we focused on the positive mutant R97M, and subsequently investigated the enzyme activity using the purified enzyme.

**Figure 6:**
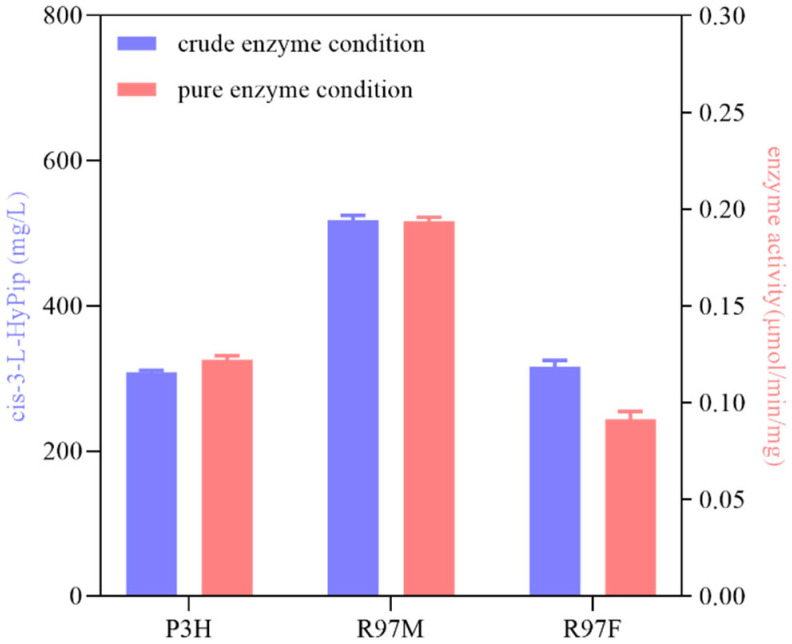
Catalytic performance of the positive mutant and the WT. The blue column represents the results under crude enzyme condition, and the corresponding blue y axis on the left represents the yield of *cis*-3-L-HyPip (mg/L). The red column represents the results under the pure enzyme condition, and the corresponding red y axis represents the enzyme specific activity (μmol/min/mg).

The enzyme activity of R97M was 1.58-fold that of the wild type (WT), reaching 0.2 μmol·min^−1^·mg^−1^ (Figure 6). Under the conditions of initial enzyme activity determination system by crude enzyme of R97M, the yield of *cis*-3-HyPip was 36% with 10 mM L-Pip as the substrate, which was 1.68 folds that of the WT. The kinetic parameters were calculated by measuring the enzymatic reaction rates of mutant R97M and WT *cis*-P3H at different concentrations of L-Pip and 2-OG. As shown in Table 2, the *K*_m_ value of L-Pip hydroxylation catalyzed by R97M was lower than that of *cis*-P3H, indicating that the affinity of R97M mutant toward L-Pip was increased compared with that of the WT. Moreover, the *k*_cat_/*K*_m_ value of R97M mutant was about 1.83-fold that of the WT. The fact of R97M showing higher affinity than the wild type was indeed consistent with the result of the virtual saturation mutagenesis. However, the actual mutation results at E112 were inconsistent with that of the virtual saturation mutagenesis. Therefore, the virtual saturation mutagenesis would provide the possibility of the mutation contributing to the function improvement and the influence of the actual mutation should be confirmed by the experimental results.

### 2.4. Molecular Function of R97 and Its Mutation in Active Pocket

**Figure 7:**
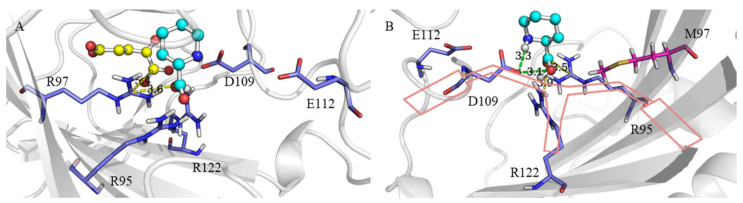
Comparison of the substrate-binding mode between the *cis*-P3H and R97M. (**A**) The “handlebar” binding mode of *cis*-P3H active pocket: salt bridge (yellow dashed line). (**B**) The “handlebar” binding mode of R97M active pocket: hydrogen bond (green dashed line) and salt bridge (yellow dashed line).

We next analyzed the conformation of the substrate-binding pockets of the WT and R97M. First, in terms of interaction, R97 in the WT formed a salt bridge with the carboxyl group of L-Pip and 2-OG. After the basic long-chain aliphatic amino acid arginine at site 97 was mutated to non-polar amino acid methionine, no salt bridge with the carboxyl group of the substrate L-Pip was found to be formed (Figure 7). It would weaken the downward force of L-Pip required to maintain a stable conformation in the active pocket. We observed that the interaction force of L-Pip was slightly balanced after the mutation, i.e., the ”handlebar” binding mode was balanced (Figure 7B). However, the salt bridge between arginine and 2-OG was also lost, which might be the reason why the mutant R97M had a lower affinity for 2-OG than the WT.

**Figure 8:**
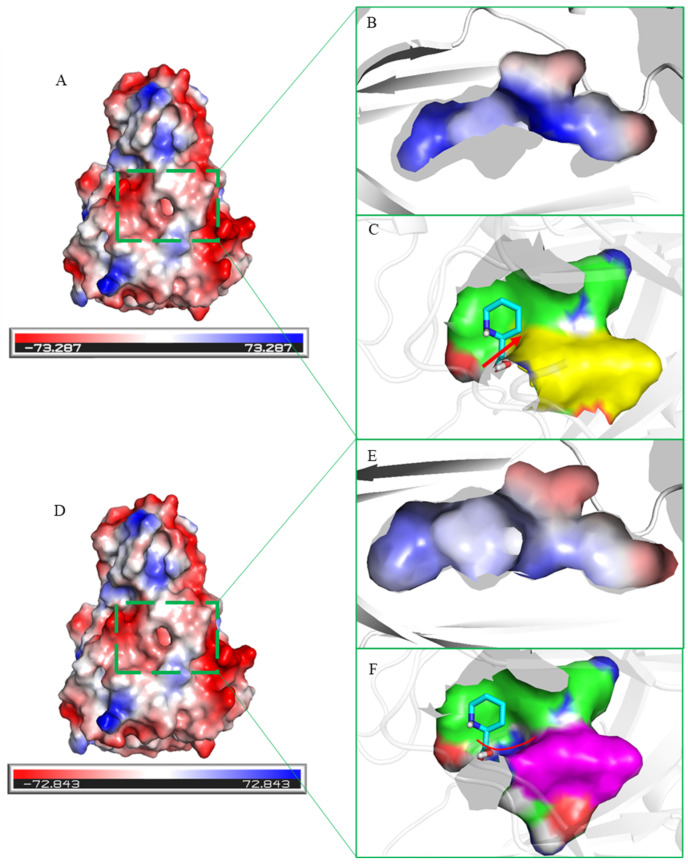
Comparison of the protein surface electrostatic potential energies between the WT and R97M. (**A**,**D**) Surface electrostatic potential energy of the WT and R97M. (**B**,**E**) Surface electrostatic potential energy near the substrate binding pocket of the WT and R97M. (**C**,**F**) Spatial conformation of the substrate binding pocket of the WT and R97M. The red arrows indicate the change of conformation of the substrate-binding pocket.

We further analyzed the protein surface electrostatic potential energy of the WT and mutant R97M (Figure 8). The substrate-binding pocket of the WT showed a positive charge. The carboxyl group of substrate L-Pip was located in the positive interaction potential region formed by the amino groups of R95, R97, and R122; the amino group of substrate L-Pip was located in the negative interaction potential region formed by the carboxyl groups of D109 and E112 (Figure 8A,D). They formed hydrogen bonds with their respective electrostatic potential regions. After R97 was mutated to methionine, the positive interaction electrostatic potential energy of the substrate binding pocket was weakened, which also confirmed the above-mentioned description regarding the interaction (Figure 8B,E).

In addition, the spatial conformation of the substrate binding pocket changed slightly. The substrate binding pocket of R97M formed a basin-like shape, different from the narrower substrate entrance of WT, which might be the reason for the increased substrate affinity of R97M (Figure 8C,F).

## 3. Materials and Methods

### 3.1. Cloning, Expression, and Purification of Dioxygenases

The coding sequences for 12 Fe(II)/2-OG-dependent dioxygenases were synthesized and cloned into vector pET-28a. The host strain *E. coli* BL21(DE3) was stored in our laboratory. Cultures were grown in the lysogeny broth medium containing 50 μg/mL kanamycin at 37 °C until the OD_600_ reached 0.6–0.8. Then, isopropyl-β-D-thiogalactopyranoside (IPTG) was added at a final concentration of 0.1 mM to induce protein expression at 17 °C for 16 h. Cell pellets were collected by centrifugation and re-suspended in 20 mM Tris-HCl (pH 8.0) containing 0.15 M NaCl. After sonication, the supernatant was used for the detection of early enzyme activity. For enzyme purification, cell debris was removed by centrifugation and His_6_-labeled *cis*-P3H was captured on Ni-NTA superfluid resin (Qiagen, Hilden, Germany). Impurity proteins were eluted with washing buffer (20 mM Tris-HCl (pH 8.0), 8 mM imidazole, 0.15 M NaCl) and *cis*-P3H was eluted with the elution buffer (250 mM imidazole). Then, the purified fractions were applied to disposable PD-10 desalting columns (GE Healthcare, Piscataway, NJ, USA) to remove the high salt content with low-salt buffer (20 mM Tris-HCl, pH 7.5, 0.1 M NaCl).

### 3.2. Structural Analysis and Virtual Saturation Mutagenesis

We used the primary amino acid sequence of *cis*-P3H to generate the structural model in the AlphaFold protein structure database (AlphaFoldDB: P96010). All docking calculations involving 2-OG and substrate L-pipecolic acid were achieved with AutoDockTools 1.5.6 [36]. For molecular dynamics (MD) simulation, the topology files of organic molecules L-Pip and 2-OG were generated online using ACPYPE Server, and the extracted *cis*-P3H structure was protonated using the H++ web server. The amber14sb force field was used to describe the dynamic behavior of atoms in MD simulation. Then the water box was set up, the TIP3P water molecules were used to solvate the system, and the total charge of the system was neutralized by adding counter ions (Na^+^ and Cl^−^). After the preparation of the simulation system, the steepest descent method was used to minimize energy. Then the NVT and NPT balance were performed. After the two balance stages, the production MD simulation of 100 ns was performed. At this stage, the simulation time step was set to 2 fs, and the trajectory file was written every 500 steps. All MD simulations were performed using the GPU version of Gromacs 2018.4.5. The trajectory file (.xtc) and molecular coordinate file (.gro) generated by MD were imported into the visualization software VMD, and the dynamic trajectory was then displayed. The relevant parameters could be set as needed to make movies or generate graphs related to dynamic distance changes.

By using Discovery Studio, virtual mutations of amino acid residues were performed on protein-ligand complexes based on interaction forces. The introduced protein structure was treated with water removal and assigned to the CHARMm force field to facilitate subsequent operations. The amino acid residues within 5 Å around the ligand and the important residue E112 were selected as the key residues, and the single point saturation mutagenesis was performed according to the operation instructions of Discovery Studio with default parameters. Three kinds of outputs can be generated: when the effect of the result is stabilizing, the mutations lead to an increase in affinity and molecular interaction; when the effect of the result is neutral, the mutations lead to no influence on affinity; when the effect of the result is destabilizing, the mutations lead to reduced affinity and weakened molecular interaction.

### 3.3. Enzyme Activity Assay

The enzymatic activity was measured in 250 μL Tris-HCl buffer (20 mM, pH 7.5) at 25 °C for evaluating the catalytic performance of dioxygenase candidates; the enzymatic activity was measured in 250 μL MES buffer (50 mM, pH 6.5) at 17 °C for further experiments with *cis*-P3H. The reaction mixture contained 10 mM substrate, 10 mM 2-OG, 10 mM L-ascorbic acid, and 0.1 mM FeSO_4_·7H_2_O. The crude or purified enzyme (100 μL) was added to the reaction mixture (150 μL), which was then incubated in a Thermomixer Comfort incubator (Eppendorf, Hamburg, Germany) at the corresponding temperature and 1000 rpm. The reactions were carried out with crude enzymes for 12 h and with pure enzymes for 15 min, respectively. The reaction was then terminated by boiling. The samples after reaction termination were analyzed by high performance liquid chromatography (HPLC). One unit of enzyme activity was defined as the amount of enzyme required to produce 1 μmol product per minute in a standard enzyme assay system.

The optimal pH for purified *cis*-P3H was determined by performing a series of equivalent enzymatic reactions in MES buffer (50 mM, pH 5.0–6.5) and Tris-HCl buffer (20 mM, pH 7.0–9.0). The optimal reaction temperature for the purified *cis*-P3H was determined by a series of equivalent enzymatic reactions carried out at 10–40 °C. The optimum temperature was 17 °C and the optimum pH was 6.5 (MES buffer) (Figure S5).

### 3.4. Determination of Kinetic Parameters

The kinetic parameters (*K*_m_ and *V*_max_) were determined in the assay mixture in a final volume of 250 μL. For L-pipecolic acid, the standard assay conditions were used, and the concentration of L-pipecolic acid ranged between 0.3 and 25 mM. Likewise, the concentration of 2-OG was varied, ranging between 0.3 and 40 mM. The assays were carried out in triplicates and the Michaelis-Menten model was fitted using GraphPad Prism [37].

### 3.5. Analysis of the Catalytic Product

The amino acid analyses were carried out using the pre-column derivatization method with Fmoc-Cl [38]. The samples containing hydroxylated products were analyzed using the Waters 2695 HPLC system equipped with a Diamonsil C18 column (5 μm, 4.6 × 250 mm). A gradient of buffer A and buffer B (50 mM sodium acetate pH 4.2/acetonitrile) was applied (gradient program, buffer A: 0–19 min 70%; 19–19.50 min 20%; 19.50–23.50 min 0%; 23.50–28 min: 70%) at a flow rate of 1 mL·min^−1^. The injection volume was 10 μL. Fmoc-Cl derivatives of amino acids and hydroxyl amino acids formed in the reaction mixture were detected spectrofluorometrically at 263 nm. The WATERS ACQUITY UPLC-MS system with chromatographic column BEH C18 (1.7 μm) was used for identification of hydroxylated products. For gradient elution, 0.1% formic acid and acetonitrile were used as mobile phases A and B, respectively. The column temperature was 45 °C, flow rate was 0.3 mL/min, and injection volume was 5 μL.

## 4. Conclusions

In this study, *cis*-P3H was engineered using semi-rational design to obtain mutants with higher catalytic efficiency toward L-Pip. Molecular docking, virtual mutation analysis, and dynamic simulations were employed to determine the key residues for site-directed saturation. The proposed “handlebar” mode of molecular interactions contributed to our understanding of the interpretation of positive mutations for increased L-Pip affinity. Consequently, a positive mutation R97M was obtained after site-directed saturation mutation, giving a *k*_cat_/*K*_m_ value of approximately 1.83-fold that of the wild type. Improvement in the catalytic efficiency of mutant R97M toward L-Pip might stem from the loss of the salt bridge between R97 and L-Pip, thereby tuning the “handlebar” balance and making slight change in the active pocket conformation, broadening the space for substrate binding. These results provide a molecular view of the binding mode toward substrates like amino acids; they also provide the basis for function-tuning engineering of *cis*-P3H and even other enzymes belonging to dioxygenase families.

## Figures and Tables

**Table 1:** Fe (II)/2-OG-dependent dioxygenase candidates for catalyzing amino acid hydroxylation.

	Enzyme	Substrate	Product	GenBank	Source	Reference
1	*trans*-P4H	L-proline	(2S,4R)-4-hydroxyproline	BAA20094.1	*Dactylosporangium* sp.	Long et al. [18]
2	*Ka*PH1			WP_025358137.1	*Kutzneria albida* DSM 43870	Xiaoran Jing et al. [19]
3	*Ka*PH2			WP_030110684.1		
4	*Ka*PH3			WP_025355730.1		
5	*cis*-P4H		(2S,4S)-4-hydroxyproline	WP_046067273	*Sinorhizobium meliloti* L5-30	Ryotaro Hara et al. [20]
6	*cis*-P3H		(2S,3R)-3-hydroxyproline	BAA22406.1	*Streptomyces* sp. TH1	Mori, H. et al. [21]
7	VioC	L-arginine	(2S,3S)-hydroxyarginine (hArg)	WP_051702361.1	*Streptomyces vinaceus* NRRL ISP-5257	Helmetag, V. et al. [22]
8	AsnO	L-asparagine	(2S,3S)-3-hydroxyasparagine	NP_627448.1	*Streptomyces coelicolor* A3(2)	Strieker, M. et al. [23]
9	AsnO D241N	L-aspartic acid	L-*threo*-hydroxyaspartic acid (L-THA)	GM869561.1	*Streptomyces coelicolor*	Strieker, M. et al. [24]
10	LdoA	L-leucine	(2S,4S)-5-hydroxyleucine	ACC80786.1	*Nostoc punctiforme* PCC 73102	Hibi, M. et al. [25]
11	IDO	L-isoleucine	4-hydroxyisoleucine	ADJ94127.1	*Bacillus thuringiensis* 2-e-2	Hibi, M. et al. [26]
12	L-lysine 4-hydroxylase (NkLH4)	L-lysine	(2S,4R)-4-hydroxylysine	AEV99100.1	*Niastella koreensis* GR20-10	Wang, Fenghua et al. [27]

**Table 2:** Kinetic parameters of *cis*-P3H and R97M.

Enzyme	Substrate	*K*_m_ (mM)	*V*_m_ (μM/min)	*k*_cat_ (min^−1^)	*k*_cat_/*K*_m_ (min^−1^ mM^−1^)
*cis*-P3H (WT)	L-Pip	10.27 ± 0.40	201.30 ± 2.00	12.23 ± 0.12	1.14 ± 0.03
	2-OG	0.76 ± 0.02	101.10 ± 1.50	6.14 ± 0.15	8.01 ± 0.38
R97M	L-Pip	6.83 ± 0.32	212.30 ± 3.50	13.82 ± 0.22	2.09 ± 0.06
	2-OG	1.46 ± 0.01	139.10 ± 1.23	9.05 ± 0.17	6.23 ± 0.32

## Data Availability

The authors confirm that the data supporting the findings of this study are available within the article and its Supplementary Materials. Derived data supporting the findings of this study are available upon request.

## Supplementary Materials

The following supporting information can be downloaded at: https://www.mdpi.com/article/10.3390/molecules28041854/s1, Figure S1: Total protein and soluble components of the expressed enzymes; Figure S2: The catalytic activity of each enzyme toward their respective natural substrates; Figure S3: HPLC chromatogram of *Ka*PH1-catalyzed reactant with L-Pip as substrate and standard (2S,5R)-5-hydroxypipecolic acid; Figure S4: LC-MS analysis of Fmoc-HyPips; Figure S5: Reaction process curve of *cis*-P3H and exploration of the optimum reaction temperature and pH of *cis*-P3H; Figure S6: Sequence alignment of *cis*-P3H (type II) and *cis*-P3H (type I); Figure S7: The results of virtual saturation mutagenesis.
