# Supplementary material for: R97 at “Handlebar” Binding Mode in Active Pocket Plays an Important Role in Fe(II)/α-Ketoglutaric Acid-Dependent Dioxygenase cis-P3H-Mediated Selective Synthesis of (2S,3R)-3-Hydroxypipecolic Acid
Source: Molecules. 2023 Feb 15;28(4):1854. doi: 10.3390/molecules28041854 (PMC9968057; doi:10.3390/molecules28041854)
Supplement: Supplementary file 1 [file molecules-28-01854-s001.zip › molecules-2207279-supplementary.pdf]

R97 at "Handlebar" Binding Mode in Active Pocket Plays an Important Role in Fe(II)/α-Ketoglutaric Acid-Dependent Dioxygenase *cis*-P3H-Mediated Selective Synthesis of (2S,3R)-3-Hydroxypipecolic Acid

Jiaojiao Guan [1], Yilei Lu [1], Zixuan Dai [1], Songyin Zhao [1], Yan Xu [1,2] and Yao Nie [1,3,*]

[1] Lab of Brewing Microbiology and Applied Enzymology, School of Biotechnology and Key Laboratory of Industrial Biotechnology of Ministry of Education, Jiangnan University, Wuxi 214122, China

[2] State Key Laboratory of Food Science and Technology, Jiangnan University, Wuxi 214122, China

[3] Suqian Industrial Technology Research Institute of Jiangnan University, Suqian 223814, China

* Correspondence: ynie@jiangnan.edu.cn

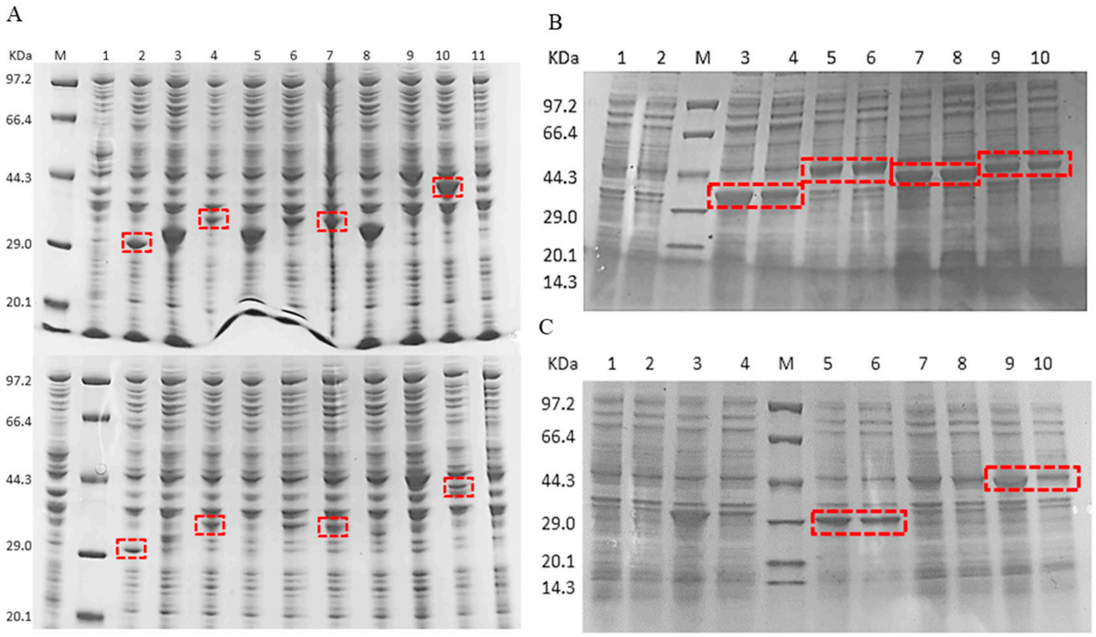

Figure S1. (A) Total protein and soluble components of *trans*-P4H (1), *Ka*PH1 (2), *Ka*PH2 (3), *Ka*PH3 (4), *cis*-P4H (7), LdoA (8), NkLH4 (10), and *E. coli* BL21(DE3) (11); (B) Total protein and soluble components of *E. coli* BL21(DE3) (1, 2), *cis*-P3H (3, 4), VioC (5, 6), AsnO (7, 8), and AsnOD241N (9, 10); (C) Total protein and soluble components of *E. coli* BL21(DE3) (1, 2), IDO (5, 6), and NkLH4 (9, 10).

The enzyme activity of the recombinant proteins was investigated with their natural substrates under the condition of initial enzyme activity determination system and crude enzyme or whole cell. The activity was determined by HPLC with Fmoc-Cl pre-column derivatization. The liquid phase chromatogram of experimental groups of *Ka*PH1, *Ka*PH2, *cis*-P4H, *cis*-P3H, VioC, AsnO, AsnOD241N, IDO and NkLH4 showed obvious new peaks compared with the blank contrast, which proving their catalytic activity (Figure S2).

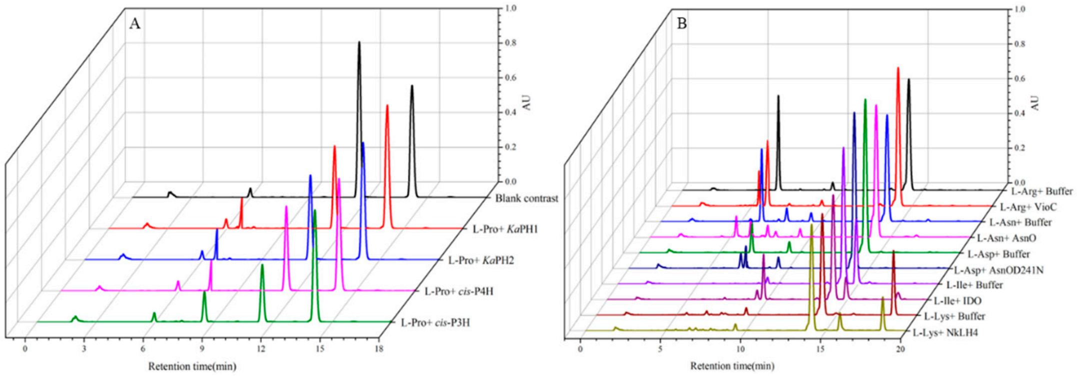

Figure S2. (A,B) The catalytic activity of each enzyme toward their respective natural substrates.

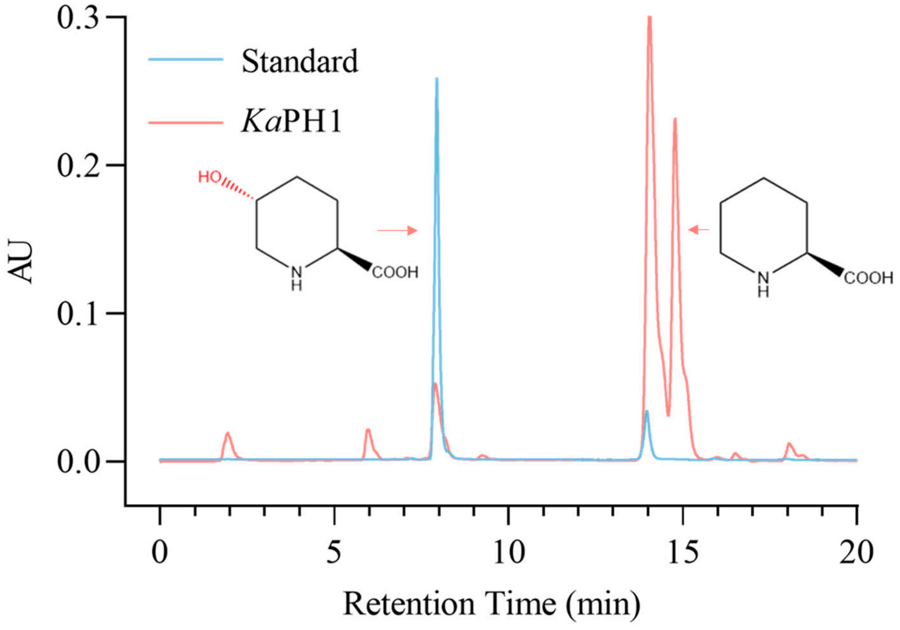

Figure S3. HPLC chromatogram of *Ka*PH1-catalyzed reactant with L-Pip as substrate and standard (2S, 5R)-5-hydroxypipecolic acid.

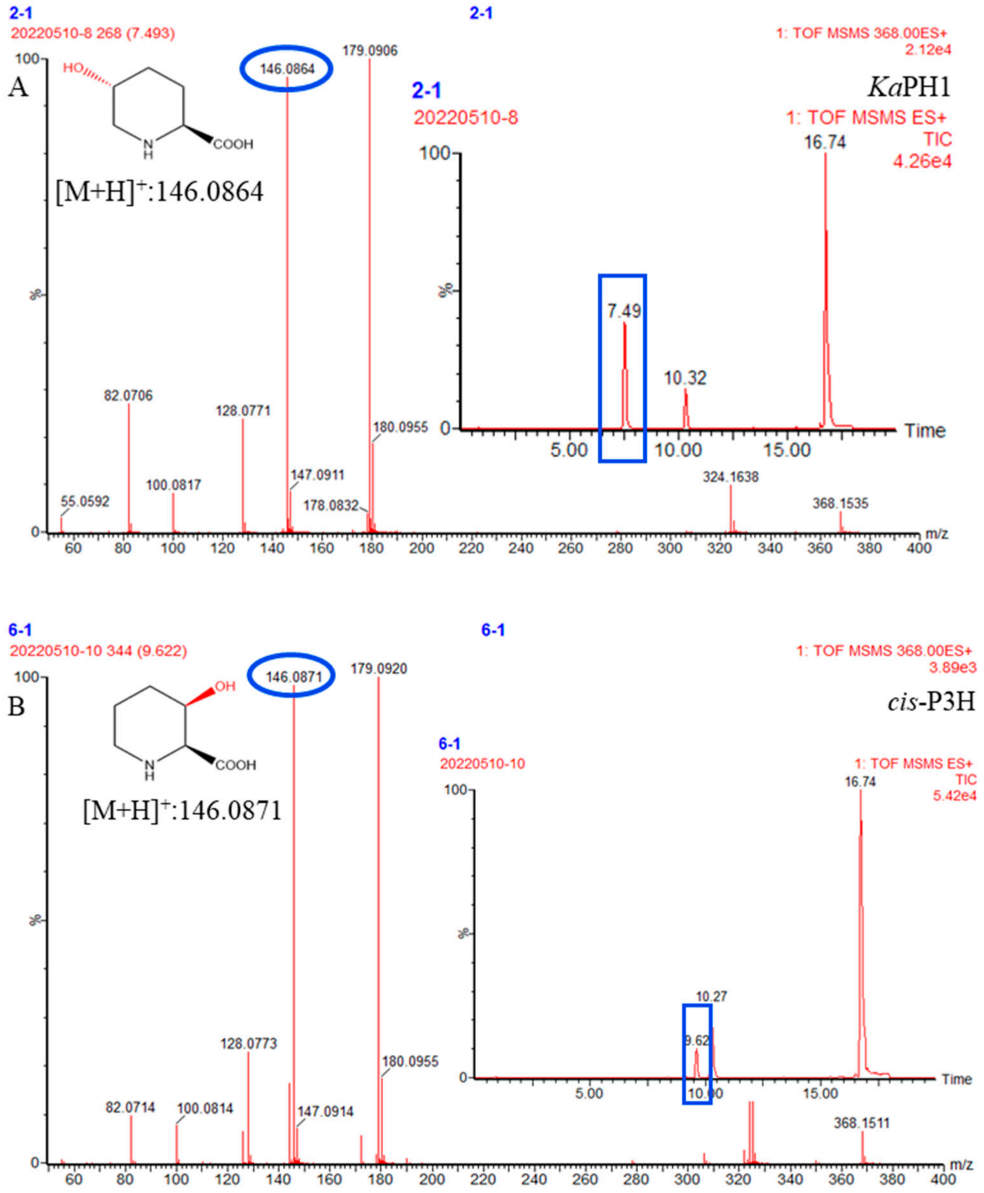

Figure S4. LC-MS analysis of Fmoc-HyPips. (A) LC-MS chromatogram of Fmoc derivative mixture of *Ka*PH1 catalyzed reaction; (B) LC-MS chromatogram of Fmoc derivative mixture of *cis*-P3H catalyzed reaction.

In the determination of enzyme activity, the reaction curve reached a linear stage within 15 minutes of measurement (Figure S5A). The enzyme activity was affected by temperature and pH, so we explored its optimal reaction conditions. The wild-type *cis*-P3H maintained more than 70% relative activity at 10-30°C, and the optimum reaction temperature was 17°C; more than 70% relative activity was maintained at pH 5.5-8.0, and its maximum activity was observed at pH 6.5 in MES buffer (Figure S5B). This laid the foundation for our subsequent research, so the reaction temperature was set to 17°C, and the reaction was carried out in MES buffer at pH 6.5.

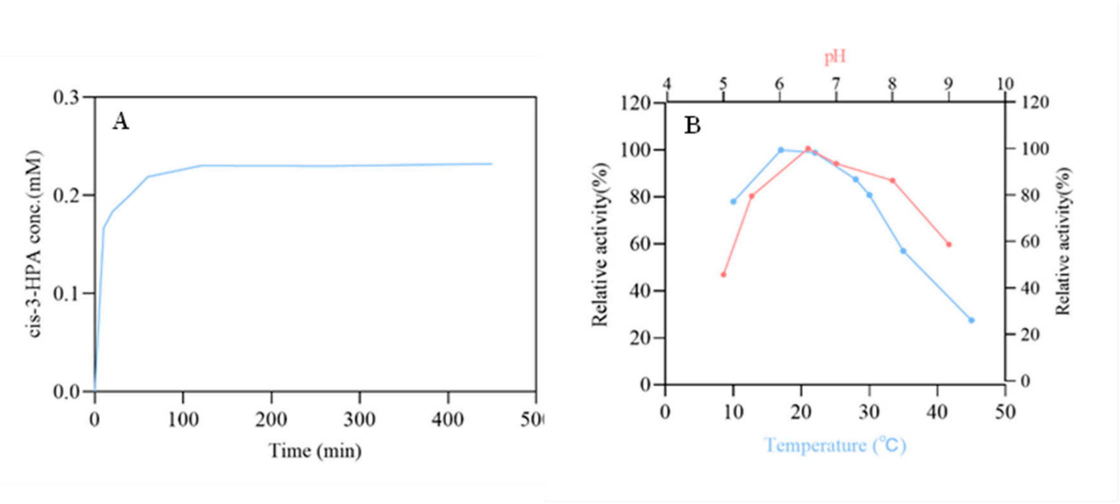

Figure S5. (A) Reaction process curve of *cis*-P3H. (B) Exploration of the optimum reaction temperature and pH of *cis*-P3H.

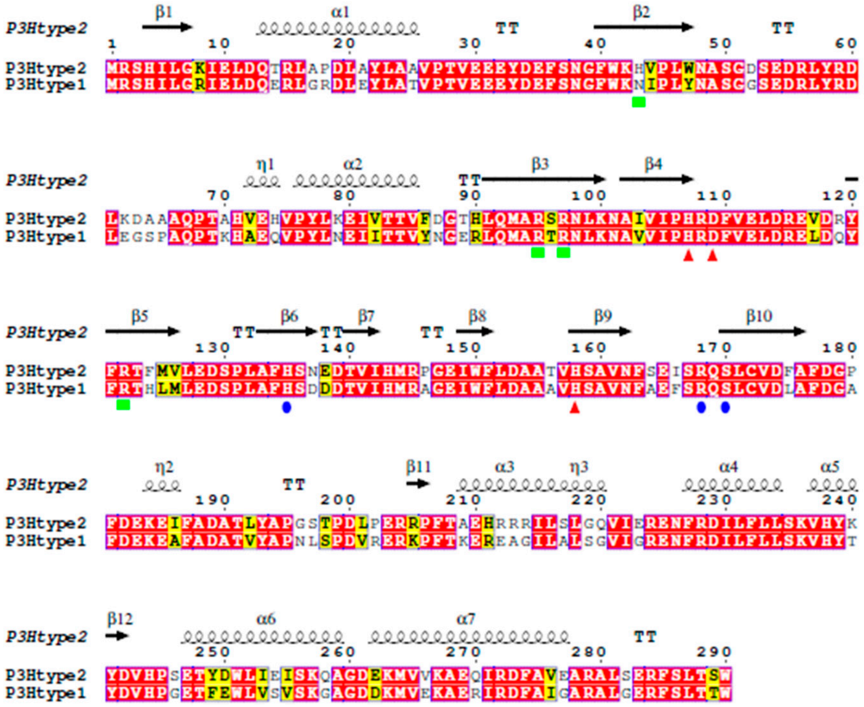

Figure S6. Sequence alignment of *cis*-P3H (type II) and *cis*-P3H (type I).

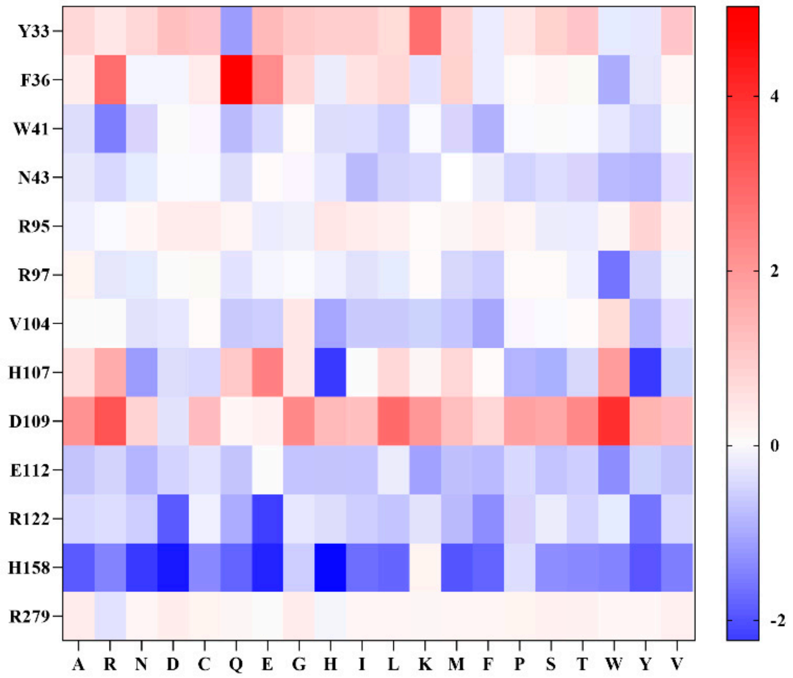

Figure S7. The results of virtual saturation mutagenesis. Red indicates low affinity and blue indicates high affinity.
